# Unraveling the harmful effect of oxidative stress on male fertility: A mechanistic insight

**DOI:** 10.3389/fendo.2023.1070692

**Published:** 2023-02-13

**Authors:** Tarique Hussain, Mahmoud Kandeel, Elsayed Metwally, Ghulam Murtaza, Dildar Hussain Kalhoro, Yulong Yin, Bie Tan, Muhammad Ismail Chughtai, Anjaleena Yaseen, Ali Afzal, Muhammad Saleem Kalhoro

**Affiliations:** ^1^ College of Animal Science and Technology, Hunan Agricultural University, Changsha, Hunan, China; ^2^ Animal Sciences Division, Nuclear Institute for Agriculture and Biology College (NIAB-C), Pakistan Institute of Engineering and Applied Sciences (PIEAS), Faisalabad, Pakistan; ^3^ Department of Biomedical Sciences, College of Veterinary Medicine, King Faisal University, Al-Hofuf, Al-Ahsa, Saudi Arabia; ^4^ Department of Pharmacology, Faculty of Veterinary Medicine, Kafrelshikh University, Kafrelshikh, Egypt; ^5^ Department of Cytology and Histology, Faculty of Veterinary Medicine, Suez Canal University, Ismailia, Egypt; ^6^ Department of Animal Reproduction, Faculty of Animal Husbandry and Veterinary Sciences, Sindh Agriculture University, Tandojam, Sindh, Pakistan; ^7^ Department of Veterinary Microbiology, Faculty of Animal Husbandry and Veterinary Sciences, Sindh Agriculture University, Tandojam, Sindh, Pakistan; ^8^ Institute of Subtropical Agriculture, Chinese Academy of Sciences, Changsha, Hunan, China; ^9^ Department of Zoology, Minhaj University, Lahore, Pakistan; ^10^ Food Engineering and Bioprocess Technology, Asian Institute of Technology, Bangkok, Thailand

**Keywords:** male infertility, ROS, oxidative stress, semen quality, steroidogenesis

## Abstract

Male infertility is a widely debated issue that affects males globally. There are several mechanisms involved. Oxidative stress is accepted to be the main contributing factor, with sperm quality and quantity affected by the overproduction of free radicals. Excess reactive oxygen species (ROS) cannot be controlled by the antioxidant system and, thus, potentially impact male fertility and hamper sperm quality parameters. Mitochondria are the driving force of sperm motility; irregularities in their function may lead to apoptosis, alterations to signaling pathway function, and, ultimately, compromised fertility. Moreover, it has been observed that the prevalence of inflammation may arrest sperm function and the production of cytokines triggered by the overproduction of ROS. Further, oxidative stress interacts with seminal plasma proteomes that influence male fertility. Enhanced ROS production disturbs the cellular constituents, particularly DNA, and sperms are unable to impregnate the ovum. Here, we review the latest information to better understand the relationship between oxidative stress and male infertility, the role of mitochondria, the cellular response, inflammation and fertility, and the interaction of seminal plasma proteomes with oxidative stress, as well as highlight the influence of oxidative stress on hormones; collectively, all of these factors are assumed to be important for the regulation of male infertility. This article may help improve our understanding of male infertility and the strategies to prevent it.

## Introduction

Male infertility is a fertility-related disorder in which a male cannot impregnate a female to achieve a successful pregnancy (1). It is a worldwide issue and contributes to 50% of infertility cases (2) and may occur for multifaceted reasons, such as disruption to the hypothalamus or pituitary function or obstruction or inflammation in the testicles, which subsequently lead to infertility. Moreover, some other conditions, such as hypogonadism, erectile dysfunction, epididymitis, congenital bilateral absence of the vas deferens, and Sertoli cell syndrome, are known to be contributing factors for male infertility (3). Most male infertility factors are idiopathic (2). All of these factors are believed to be directly or indirectly involved in the production of oxidative stress. Reactive oxygen species (ROS) are the active oxidative metabolites that are responsible for producing oxidative stress and are also a prominent cause of male infertility (4, 5). Overwhelming oxidative stress may influence the reproductive system, as well as aspects of the semen, such as sperm concentration, motility, and morphology, thus causing a deterioration in semen quality, resulting in a poor conception rate (6). It has been noted that oxidative stress is involved in diseases that affect male fertility status (7).

The sperm plasma membrane contains polyunsaturated fatty acids, which make it more delicate and vulnerable to oxidative damage, and eventually spermatozoa lose the capacity to fertilize. Moreover, fragmented DNA may impair the paternal genetic ability to develop embryos (5). ROS consist of one or more unpaired electrons, which are capable of damaging lipids, carbohydrates, DNA, and amino acids (8). Interestingly, ROS exist in three forms: primary, secondary, and tertiary. Not all ROS are free radicals (5, 9); however, the physiological concentration of ROS plays a pivotal role in sperm capacitation, hyperactivation, and other acrosomal changes (10). Evidence has indicated that 30–80% of male-related fertility issues are a result of ROS-triggered sperm damage (11–13).

Advanced proteomic tools allow the characterization of semen profiles by applying mechanistic approaches and are helpful for detecting proteins and their underlying molecular mechanisms, which can predict the significance of male fertility-related hindrances (13). Increasing knowledge in this area permits easy understanding of the seminal plasma and sperm proteins and allows the identification of differences between fertile and infertile men (14). Previous literature revealed the relationship between oxidative stress-potentiated male infertility and the sperm and seminal plasma protein profile; alterations to the expression and function of proteins may be evident at sperm maturation. Further studies are needed to identify the pathologies linked to male infertility on molecular and proteomic levels. The impact of oxidative stress has well been documented in male infertility, although limited literature exists about the relationship between oxidative stress and the proteomic profile of human ejaculation. The current literature regarding human infertility reveals the association between oxidative stress and the proteomic profile (15–17). Additionally, further studies based on proteomic profiles have documented poor semen quality, which is influenced by oxidative stress (18, 19).

Male infertility cases can be diagnosed through the evaluation of basic semen characteristics, such as liquefaction time, sperm count, motility, morphological features, and sperm viability. However, the WHO has set some guidelines or reference values for sperm abnormality, alteration of sperm concentration, motility, and morphology by which the fertility status of humans, as well as animals, can be assessed (20, 21). Numerous advanced tools can be applied to figure out the possible causes of infertility, based on the detection of free radicals, antioxidant capacity analysis, sperm DNA oxidation, DNA compaction, apoptosis, the presence of anti-sperm antibodies, and genetic testing (22). Elevated concentrations of ROS have been reported in infertile human patients with DNA damage and unstable chromatin packing (5). Sperm DNA damage is a biomarker for the loss of cellular integrity, which is associated with a decline in semen quality, and is thus regarded as a cause of infertility in many humans (23, 24). Assisted reproductive biotechnology using spermatozoa with fragmented DNA is more susceptible to lower fertilization and pregnancy rates, abnormal embryonic development, and an increased risk of miscarriage, congenital defects, and other anomalies that occur during childhood (25, 26). The amount of DNA fragmentation is a viable indicator of assisted reproductive outcomes in idiopathic infertile couples. However, increased sperm DNA fragmentation has been associated with lower birth weight after IVF treatment (24). Our main purpose when designing this review was to elaborate on the role of mitochondria, the cellular response in fertility-related problems, and the interaction of seminal plasma proteomes with oxidative stress and highlight the influence of oxidative stress on hormones.

## ROS and mitochondria function

A higher level of ROS induces oxidative stress caused by oxidants in germ cells. Mitochondria are key organelles; at low levels, ROS maintain redox balance. Excessive levels of ROS potentiate lipid peroxidation events, which inhibit small molecules of aldehydes, such as acrolein, malondialdehyde, and 4-hydroxynonenal (4-HNE). These molecules bind with protein sites at susceptible histidine, lysine, and cysteine residues on targeted proteins (27). The activity of these proteins impairs electron flow towards the mitochondrial electron transport chain and generates free radicals that are responsible for the production of more aldehyde products (27). Any factor that influences germ cells through the production of oxidants by way of oxidative phosphorylation can cause oxidant cascades. Oxidative stress occurs for several reasons, such as a lack of antioxidants, ionization radiation, leukocytes, obesity, smoking, reproductive tract infections, and pesticides. A positive relationship between spermatozoa consisting of polyunsaturated fatty acids and free radicals has been well established.

**Figure 1:**
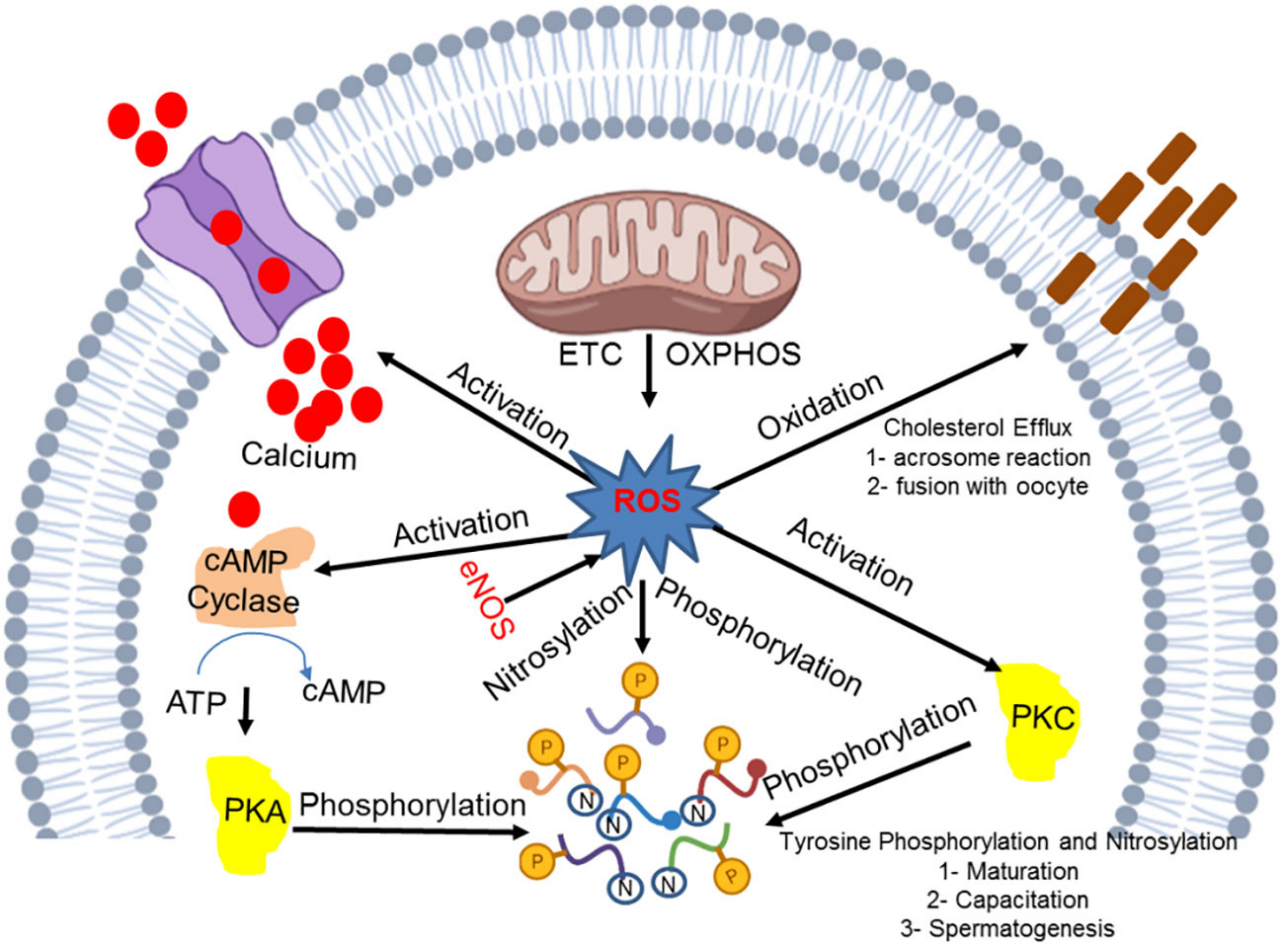
Molecular insight into the ROS and RNS mechanism in the development and functional integrity of spermatozoa.

Mitochondrial ROS production is an essential process for inducing intrinsic apoptosis. A huge number of spermatozoa eventually undergo apoptosis, while the limited number that remain are pivotal for the successful continuation of the fertilization process. Lipopolysaccharide (LPS), a bacterial endotoxin, is known to induce apoptosis (28) in a large number of spermatozoa. Apoptosis is an essential process for the continuation of life as macrophages and neutrophils rarely exert phagocytosis to eliminate dead spermatozoa. It has been noted that the apoptotic process can be completed irrespective of the activation of inflammation, cytokines, and ROS production. However, leukocyte infiltration causes a damaging effect due to the occurrence of an inflammatory response that repeats after a vasectomy or during a sexual act. These sperms induce a response, which may be reversed in the presence of phosphatidylserine (an apoptosis marker), that subjects gametes to phagocytosis. The ROS and RNS mechanism that is crucial for the basic development correction and functional activity of spermatozoa is displayed in **Figure 1**.

Realistically, spermatozoa undergo apoptosis due to the activation of an enzyme that cells utilize for their survival, known as phosphatidylinositol-4,5-bisphosphate 3-kinase (PI3K) (29). As the activation of the PI3K signaling pathway takes place, it phosphorylates downstream kinases, such as AKT (Protein kinase B), thus stimulation of these pathways makes gametes active and viable. AKT targets downstream proteins, such as BCL_2_-related death promoter (BAD), which, once dephosphorylated, form pro-apoptotic pores in the mitochondrial membrane and foster pore formation with BAK/BAX (30). Once the basic mechanism of sperm apoptotic origin is understood, it is necessary to understand the underlying mechanism that promotes PI3K activity. Of note, spermatozoa contain several pro-survival hormonal receptors, such as prolactin (31) and insulin, once they are stimulated by their respective ligands, which makes their continuous survival sustainable. By contrast, if the PI3K inhibitor wortmanin is used then gametes promptly promote mitochondrial ROS generation, rendering cells more susceptible to apoptosis (29).

## ROS and the capacitation process

ROS are stimulated by several mechanisms that are based on the activation of adenylyl cyclase activity (32), which in turn stimulates protein kinase A (33, 34). H_2_0_2_ plays the key role during capacitation of mediating the processes of phosphorylation and capacitation, and this has been well reported in suspensions of hamster, bovine, and human sperm (32). Likewise, exposure of the spermatozoa to synthetic oxidized conditions induces the extracellular generation of ROS through glucose oxidase or xanthine oxidase systems and the initiation of the capacitation process. Tyrosine phosphorylation can be attenuated by the induction of catalase in several species (32). However, ROS-generated leucocytes contribute to human sperm capacitation and may reverse in the presence of seminal plasma antioxidants (35). The profound function of H_2_0_2_ is illustrated by catalase, which restores the spontaneous induction of tyrosine phosphorylation in capacitating mammalian spermatozoa, and hence, reduces functions, such as hyperactivation, acrosomal exocytosis, and sperm-egg fusion; all of these steps are achieved following capacitation (36). A variety of ROS sources have been used for the activation of capacitation processes, such as superoxide anion, nitric oxide, and peroxynitrite (37). It has been noted that a huge interconversion of ROS occurs during sperm capacitation and any ROS can take part in it. If the potential of oxidative metabolites displays a crucial role in capacitation then the regulators will be H_2_O_2_ and peroxynitriate. Peroxynitriate is responsible for producing a variety of capacitating spermatozoa features, e.g., suppression of tyrosine phosphate activity (38).

**Figure 2:**
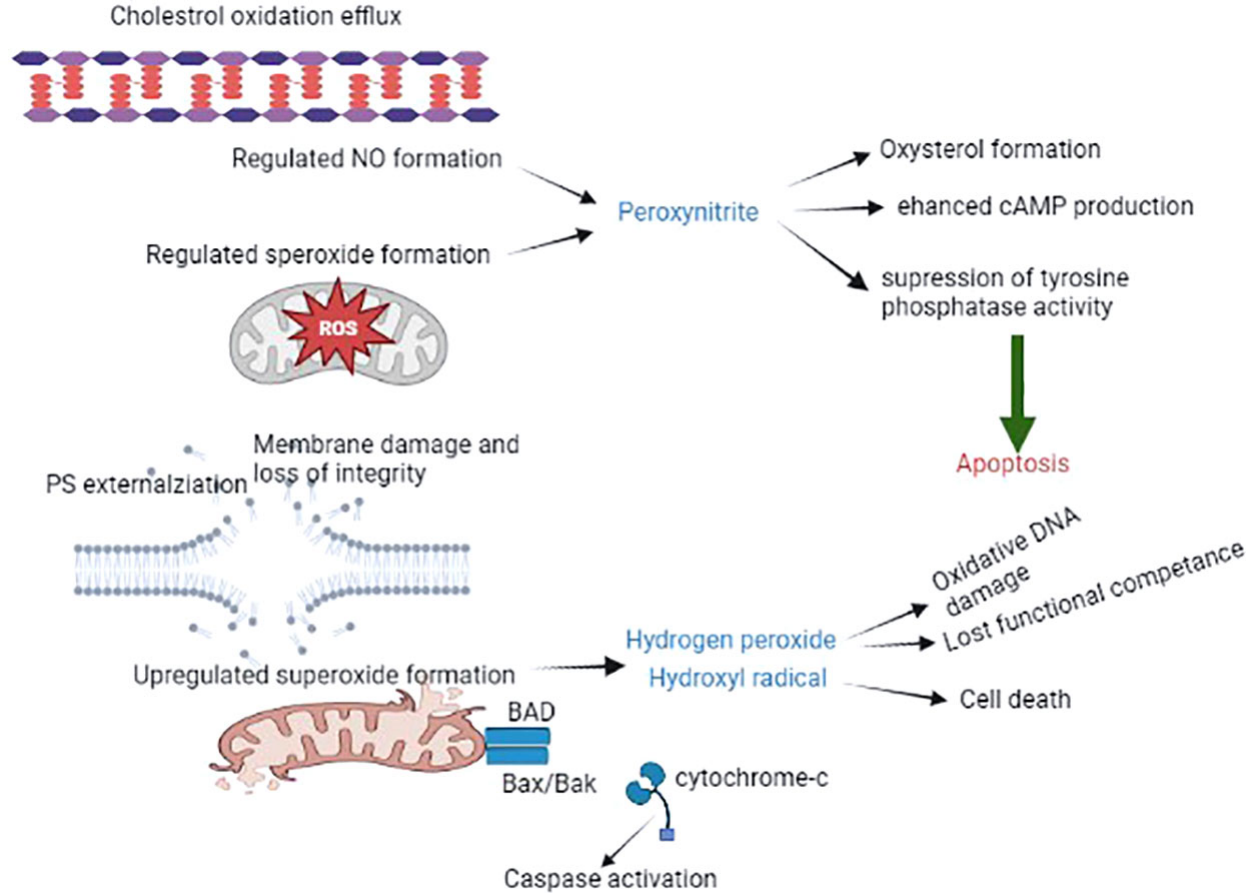
The spermatozoa capacitation process and apoptosis. The schematic diagram illustrates the accelerating production of ROS (mainly ONOO), resulting in the generation of oxysterol, which helps to eliminate cholesterol from the plasma membrane, leading to the promotion of membrane fluidity and other alterations, such as tyrosine phosphatase suppression and enhanced cAMP activity. This process eventually leads to capacitated spermatozoa. The absence of fertilization results in the generation of oxysterol and lipid aldehydes, which trigger apoptosis, resulting in increased mitochondrial superoxide production, lipid peroxidation, cytochrome c release, caspase activation, phosphatidylserine exposure, oxidative DNA fragmentation, and ultimately death.

The positive impact of ROS generation and capacitation has been reported in the female reproductive tract. Spermatozoa can only produce excessive ROS once they are released by the oviductal epithelium, immediately before the site of fertilization. In this scenario, every spermatozoon is briefly exposed to ROS and prepares itself for fertilization. In case fertilization by spermatozoa does not occur, spontaneous free radical production leads to overcapacitation and ultimately induces oxidative stress. Eventually, this leads to the production of lipid aldehydes, which initiate ROS-mediated peroxidation and subsequently trigger apoptosis (39, 40). Sperm capacitation towards apoptosis may assist in the long-term storage of spermatozoa to sustain sperm capacitation for a longer period. The reality is that several domestic species of spermatozoa undergo capacitation-like changes that lead to oxidative and stress-related cryopreservation, and this may be a potential factor facilitating the longevity of these gametes prior to insemination (41). The best way to ameliorate oxidative stress-induced cryopreserved spermatozoa is through the addition of antioxidants, such as lycopene, cysteamine, melatonin, vitamin E, and resveratrol (42), which are widely used due to their significant impact. Molecular insights into the spermatozoa capacitation process and apoptosis are depicted in **Figure 2**.

## Oxidative insult and male hormones in reproduction

The occurrence of oxidative stress depends on either the overproduction of ROS or depletion of antioxidants, which may result in lipid peroxidation in Leydig cells and germ cells and is detrimental to lipoproteins, protein aggregation and fragmentation, and steroidogenic enzyme inhibition (43). The prevalence of OS in the testicles results in declining testosterone production due to injury of the Leydig cells or other endocrine structures, such as the anterior pituitary (44, 45). It is notable that the physiological production of hormones also produces ROS that are mainly derived from mitochondrial respiration and catalytic reactions of the steroidogenic cytochrome P450 enzymes (46). In this way, the production of ROS suppresses the substantial production of steroids and is deleterious to the mitochondrial membranes of the spermatozoa (47). OS is associated with a higher number of immature spermatozoa through an indirect effect on male hormone production, which is associated with spermatogenesis (48, 49).

It has been noted that hormones, such as follicle stimulating hormone (FSH), luteinizing hormone (LH), testosterone, estrogen (E2) and prolactin (PRL), may regulate seminal total antioxidant capacity (TAC) (50, 51). An association between PRL or free thyroxine T4 (fT4) and a negative correlation of gonadotropins or gonadal steroids with TAC have also been observed (52). It is believed that some hormones, such as testosterone and melatonin (MLT), may increase antioxidant capacity to defend sperm and other testicular cells from the detrimental effects of ROS (53, 54). Other hormonal metabolites, such as dehydroepiandrosterone (DHEA), increase cellular antioxidants through an exact mechanism that remains elusive (55). In infertile men, direct and indirect connections between testosterone and antioxidant levels and between testosterone and zinc have been documented (51, 56). Coenzyme Q10 (CoQ10) may reduce the concentrations of FSH and LH (57). The negative association has been exhibited in serum concentrations of testosterone, E2, fT4, and sperm DNA fragmentation (58, 59). The suppression of antioxidants might influence triiodothyronine (T3), thyroxine (T4), and neurotransmitter noradrenaline and elevate sperm DNA fragmentation (60). The administration of highly purified FSH to idiopathic infertile men reduces ROS production (61) and sperm DNA damage (62). However, it has been found that testosterone may trigger DNA fragmentation and germ cell caspase activities in Sertoli cells (63), and a longer antioxidant effect may modulate FSH, testosterone, and inhibin B concentration (64).

As discussed above, excessive ROS influences the hypothalamic-pituitary adrenal axis (HPA) and in turn releases corticosterone and cortisol in animals and humans, which induces stress. The release of stress hormones impairs crosstalk between HPG and HPA axes, disrupting LH production by the anterior pituitary gland. Reduced levels of LH are unable to activate Leydig cells, which produce testosterone. Reduced FSH weakens the production of androgen-binding protein (ABP) by the Sertoli cells, and hence, collectively reduces the level of circulatory testosterone due to abundant OS. ROS influences the HPT axis to diminish T3 and T4 secretion. Reduced T3 levels of steroidogenic acute regulatory protein (StAR) mRNA, protein in Leydig cells, and testosterone production occur (65). The overproduction of OS reduces insulin production by the pancreas, which exerts a detrimental effect on T3 release by the thyroid gland, and therefore, testosterone biosynthesis occurs. During OS production, testicular E2 and inhibin are largely secreted, which suppress testosterone production. After OS production, aromatase activity is enhanced, which leads to higher E2 production. Further, exposure to ROS promotes PRL release by the anterior pituitary gland, which results in a reduction in the release of GnRH. In summary, it has been observed that OS disrupt hormonal communication in different ways. Hormones play a key role in the functionality of the male reproductive system. ROS affect testosterone production, which in turn influences spermatogenesis. Moreover, OS also affects male reproductive behavior by interfering with testosterone production, eventually causing infertility.

## Cellular response against inflammation and infertility

Inflammation is a natural defense against foreign invaders that causes cellular injury and subsequently results in the restoration of tissue function (66, 67). It has been noted that an inflammatory response is established due to the excessive production of prostaglandin E2, cytokines, and nitric oxide (NO) by macrophages and other inflammatory cells (68). Some evidence suggests that inflammation may affect steroidogenesis and spermatogenesis. A sudden decline of blood testosterone and luteinizing hormones have also been noted with inflammation (69). A study was conducted in which lipopolysaccharide (LPS) was used to stimulate an inflammatory response; a considerable reduction in testosterone was observed. However, a lower response of steroidogenesis was reported and known to be called steroid acute regulatory StAR proteins (70). Evidence has revealed that inflammation increases spermatogenic arrest and inhibits the sperm maturation process (71). The epididymis is another target of inflammation due to testicular attacks. Importantly, inflammation is triggered by leukocytes that infiltrate semen and secrete anti-sperm antibodies. The inflammation reaction promotes rigidity of the sperm flagella membrane by reducing the lipid content of the membrane. Thus, the inhibition of sperm motility may result in sperm agglutination and asthenospermia. Additionally, it causes defects in acrosome reaction, which prevent sperm penetrating the oolemma. Moreover, it suppresses DNA integrity due to the increased number of apoptotic sperm cells (5).

Previous literature indicate a relationship between oxidative stress and inflammation. The inflammatory response has been documented in semen due to elevated levels of ROS in infertile men (72). Moreover, invading bacteria generate ROS by themselves, whereas leukocytes are considered to be the essential player of seminal ROS (8). Leukocytes increase ROS production in two ways, one direct and one indirect; the indirect source involves the release of inflammatory cytokines, which increase the level of ROS. A direct increase in ROS is achieved through the activation of phagocytosis. These oxidants harm the spermatozoon membrane, resulting in an oxidative burst in which the oxidant/antioxidant ratio is severely disrupted. This scenario even occurs when pathogens are successfully executed (69).

Cytokines are polypeptide proteins that are attributed to immune response, cellular growth and differentiation, inflammation, etc. In the male reproductive tract, cytokines are secreted by the testes and are responsible for germ cell proliferation and the differentiation of mesenchymal cells and take part in steroid anabolism (8, 73). A growing number of studies have revealed that ROS and cytokines interplay with each other in a complex manner. ROS increase cytokine production, while a few cytokines regulate the pro-oxidant and antioxidant system and ROS production (5, 74, 75). Many studies have described the relationship between cytokines and ROS. For example, increased concentrations of IL-6 and IL-8 trigger the peroxidation process, influencing sperm functionality and eventually causing infertility during male reproductive tract inflammation (5). The limited concentration of cytokines plays an essential role in the male gonad’s function and seems to be present in seminal plasma (76). A vast network of cytokines, chemokines, and growth factors, along with their soluble receptors and antagonists and other factors, were investigated in human semen (76, 77). Human semen also secretes tumor necrosis factor α (TNF-α), interleukins, IFN-γ, and some of their soluble receptors, which are present in immune cells, mesenchymal cells, Sertoli cells, and spermatogonia. The physiological concentration of cytokines (IL-6, IL-8, and TNF-α) has been reported in human semen (76, 77). Cytokines are not directly involved in apoptosis, but TNF-α, TGF-β2, and TGF-β3, in addition to testosterone, are capable of regulating spermatogenesis (78, 79). However, TGF-β plays various roles in cellular functions, including the secretary function of Leydig cells, Sertoli cells, the biological development of testes, and spermatogenesis intensity (80). As discussed earlier, sperm consists of an array of cytokines and immune factors, although their effect on semen quality and sperm function parameters needs to be debated (81, 82).

## Oxidative insults, cellular defense, and male reproduction

It has been reported that increased oxidative stress can be implicated in various pathogenic conditions, such as inflammation, ischemia, and heat stress, which suggests it plays an important role in male infertility (5, 83). Spermatozoa are easily targeted by oxidation. Spermatogenic cells eliminate oxidative DNA by apoptosis through p53-dependent and -independent mechanisms (84), showing that higher activity may lead to male infertility (85). However, redox-sensitive proteins are the most susceptible to ROS and are regarded as ROS potent targets under oxidative stress.

The cellular antioxidant defense neutralizes ROS (superoxide anion radicals), such as superoxide dismutase (SOD) and glutathione peroxidase (GPX). Further, a detailed review of an antioxidant enzymatic system in male reproduction has been discussed (86, 87); only the ameliorative effects of ROS in male reproductive anomalies have been described in this Review. SOD is an enzyme that converts superoxide radicals into hydrogen peroxide, which halts the deleterious effects of a radical chain reaction at the initiation stage (88). Copper-zinc sodium dismutase encoded by SOD1 is mainly present in the cytoplasm, and partly in mitochondria. Although SOD1-deficient females are infertile, SOD1 is not associated with anomalies that hamper male fertility (89). Moreover, SOD1 enzyme deficiency may induce testicular atrophy and confer proneness to heat stress (90). Sperm numbers following higher incidences of lipid peroxidation products were reduced in aged SOD1-deficient mice compared with wild-type mice (91), while the fertilizing ability linkage was not found in mice. Manganese sodium dismutase, a mitochondrial isoform, works under the influence of oxidative stress and inflammation. The deficiency of this enzyme is lethal once the fetus/infant is born (92). Moreover, transgenic mice may exhibit a higher expression of SOD2 and are infertile; the underlying mechanism of this condition is unknown (93). SOD3 is an extracellular SOD that is present at high levels in epididymis fluid and at low levels in spermatogenic cells (94). SOD3 knockout mice do not show any prominent phenotypic alteration in male reproduction, although the presence of SOD3 in the penis has been associated with increased erectile function in aged mice (95). The superoxide radical rapidly reacts with nitric oxide to form peroxinitrite and higher levels of SOD3 in blood plasma enhance the half-life of nitric oxide and ultimately promote erectile functions. Conversely, excessive SOD activity has been linked with human sperm movement anomalies (96) that may eliminate superoxide, suggesting that SOD plays an important role in sperm movement. As a result, both the source and the underlying mechanism determine whether superoxide has a beneficial or detrimental effect on reproductive function.

It has long been known that a variety of sources of hydrogen peroxide production *via* enzymatic and non-enzymatic reactions exist and their successful elimination is carried out by glutathione peroxidase (GPX), catalase, and peroxiredoxin (PRDX). GPX demonstrates this by catalyzing the reduction of different peroxidases through the transfer of electrons from glutathione (97), while the functions of each member and gene family are different and complex (98). Peroxiredoxins (PRDXs) catalyze the reductive removal of hydrogen peroxide with the help of thioredoxin (Trx), not glutathione, as it donates an electron (99) and also has multifaceted functions in redox reactions consisting of ROS signaling.

## Oxidative stress in seminal plasma and sperm

Free radicals or ROS are oxygen-based centered radicals with one or more unpaired electrons (100). Examples of free radicals and non-radicals are hydroxyl, superoxide, peroxyl, and lipid peroxyl, while the non-radicals consists of singlet oxygen, hydrogen peroxides, hypochloric acid, lipid peroxide, and ozone (101). The most important ROS from sperm are hydroxyl radicals, superoxide anion, and hydrogen peroxide. Owing to their high reactivity in nature, they possess very short half-lives, i.e., nanoseconds (10−9 s) for hydroxyl radicals and milliseconds (10−3 s) for superoxide anion; therefore, they react at their generation site (102, 103). Moreover, when the dismutation of superoxide anion takes place, it causes the formation of hydrogen peroxide, which is almost a weak free radical (104, 105). Once the superoxide and hydrogen peroxide are produced, they undergo different cellular reactions and then transform into highly powerful hydroxyl radical free radicals *via* the Fenton and Haber–Weiss reaction (106). In addition to that, superoxide anion interrelates with nitric oxide and forms a peroxynitrite. Nitric oxide is also regarded as a reactive free radical with an odd number of electrons (105, 107). The most critical sources of ROS production in males are leukocytes and immature spermatozoa (85). Leukocytes, especially neutrophils and macrophages, have been linked with excessive ROS formation, which causes sperm dysfunction (101, 108).

## Oxidants and semen parameters

**Figure 3:**
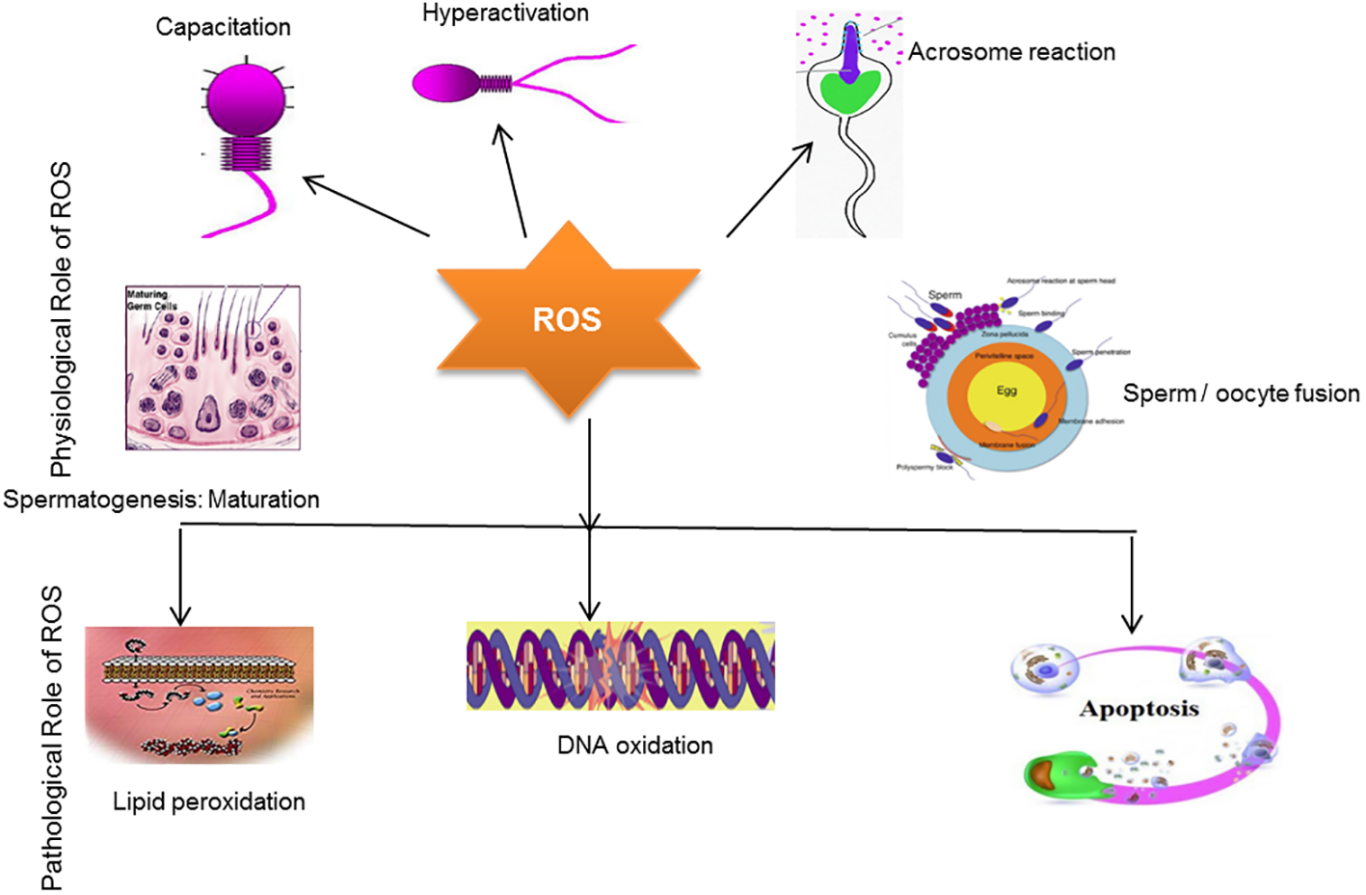
The positive and negative effect of ROS in male infertility. The schematic diagram demonstrates that a limited concentration of ROS plays a key role in sperm capacitation, sperm hyperactivation, acrosomal reaction, sperm maturation, and sperm/oocyte fusion processes. Conversely, overproduction of ROS induces oxidative stress, which ultimately damages the sperm membrane, causing lipid peroxidation and DNA oxidation, which eventually induce apoptosis, resulting in infertility.

It is well known that limited concentrations of ROS are essential for spermatogenesis. ROS generation in the context of antioxidants is necessary for spermatogenic processes to occur. Antioxidants, such as hydrogen peroxide, contribute to the sperm capacitation process and thus help spermatozoa bind to the zona pellucida and fertilize the egg (9, 83). Notably, catalase causes the decomposition of H_2_0_2_ and also maintains sperm motility (5). This relationship between H_2_0_2_ and catalase balances redox status and abrogates oxidative stress. Free radicals are the byproducts of oxidative metabolism in mitochondria. In response to these reactions, oxygen reduction occurs in mitochondria (5). Normally, mitochondria are located in the midpiece of the sperm, and studies have indicated that mitochondrial DNA is more vulnerable to mutation than nuclear DNA; therefore, it elevates ROS production (109). Enhanced ROS production has been associated with the stimulation of cytochrome-c, a protein involved in apoptosis (programmed cell death), and is reported in males with infertility problems (110). Previous studies have highlighted that the generation of mitochondrial-mediated ROS production is deeply involved in DNA damage (111). DNA damage by ROS may subsequently lead to poor *in vitro* blastocyst formation (112). Moreover, the sperm membrane mainly consists of polyunsaturated fatty acids, which play a key role in membrane fusion (9). However, seminal fluid is an essential source of antioxidants in semen, as the spermatozoa’s lack of cytoplasm and DNA compaction mean there is little space for antioxidant enzymic translations (113). As aforementioned, the fragile structure of the sperm membrane can be easily targeted by ROS, which ultimately affects sperm motility (114). The positive and negative effect of ROS on male infertility is illustrated in **Figure 3**.

## Seminal plasma proteomes and oxidative stress

A variety of proteins have been proposed to be the possible markers of oxidative stress (OS) damage. Wang and colleagues suggest that the decline of DJ-1 protein contributes to the suppression of OS triggered by endocrine disruptors, a proposed marker of OS in asthenozoospermic patients (19). Herwig and colleagues observed that tubulin folding cofactor β and higher levels of α-1 chymotrypsin and aldose reductase are associated with OS in patients with idiopathic oligo-astheno-teratozoospermia (18). Another study indicated the increased expression of prolactin-triggered protein, which is related to OS damage and poor sperm quality (16). Intasqui and colleagues showed that overexpression of mucin 5B in normozoospermicmen correlated with increased seminal lipid peroxidation levels, suggesting that this protein contributes to sperm transport alteration in both sexes; therefore, it could be a marker of lipid peroxidation resulting from OS (115). Of note, OS-augmented modulation of the seminal plasma proteome does not normally occur on large scales in infertile patients, but it also prevails in fertile subjects. In a current study, fertile patients with enhanced OS exhibited overexpression of proteins attributed to stress response, such as haptoglobin (HP), peroxiredoxin 4 (PRDX4), and protein S100 calcium-binding protein A9 (S100A9). Specifically, HP and PRDX4 exert antioxidant properties, thus their overexpression is known to be involved in the scavenging effect against the overproduction of ROS. The S100A9 protein possesses pro-inflammatory activity; its overexpression along with C3 complement shows the inflammatory state caused by OS (116). The overexpression of seminal plasma proteome due to oxidative stress in patients is presented in **Table 1**.

**Table 1:** Overexpressed seminal plasma proteins in patients showing signs of oxidative stress.

Seminal plasma proteins	Functions	References
Aldose reductase	Converts glucose into sorbitol in the polyol pathway (glucose metabolism)	(18)
α1-chymotrypsin	Proteolytic activity towards chymotrypsin-specific substrate N-Succinyl-Ala-Ala-Pro-Phe-p-nitroanilide and releases in granulocytes	(18)
DJ-1	DJ-1 activation causes catalysis of ROS. Once activated, suppress DJ-1, resulting in the removal of NFκB signal	(19)
Haptoglobin	late positive acute-phase protein of inflammation	(116)
Mucin 5B	Promotes SP viscosity and is associated with inflammation, hypoxia, and OS	(115)
Peroxiredoxin 4	Related to a family of peroxide-degrading enzymes, contributes to cellular OS control	(116)
Prolactin-induced protein	Extracellular matrix protein that may regulate tissue responses to inflammation	(16)
Protein S100A9	Pivotal in cell differentiation and OS response	(116)
Tubulin-folding cofactor β	Participates in the development of α/β-tubulin heterodimers, essential for the normal growth of mammalian cells. Serves in the development of hypoxic-ischemic injury	(18)

NFκB, nuclear factor kappa light chain enhancer; OS, oxidative stress; ROS, reactive oxygen species; SP, seminal plasma; S100A9, S100 calcium binding protein A9.

Lifestyle and diseases may trigger seminal plasma OS. Obesity, alcohol abuse, cigarette smoking, and heavy metals have been firmly linked with OS. Moreover, environmental factors, such as heavy metals, also contribute to excessive ROS. Additionally, ROS occur as a result of various diseases, such as accessory gland infection/inflammation (MAGI) and varicocele (117). At present, irrespective of varicocele, few studies have reported SP proteome modulation in patients. Moreover, there is no literature about the differential expression of proteins with and without increased ROS, and few proteins have been suggested as disease markers. However, intelectin 1 overexpression has been observed in asthenozoospermic patients with OS, revealing the possible existence of genital tract infection. Likewise, a study reported that alcohol dehydrogenase overexpression contributed to alcohol metabolism and aminolevulenic acid dehydratase overexpression indicated exposure to lead. Thus, it proves that lifestyle and environmental factors have a detrimental effect on sperm quality due to the overproduction of free radicals (19). Further detailed information regarding the involvement of male infertility problems is well illustrated by (118).

## Evidence of sperm transcriptomic profile and oxidative stress

Transcriptionally sperm cells are energetic; RNAs are presumed to be involved in spermatogenic events (119). It is thought that sperm RNAs are linked to several functions, including fertilization (119, 120). Further, RNAs in sperm are known to be indicators of sperm quality index (121–123) and fertility (120, 124, 125). Interestingly, sperm consists of coding and non-coding RNAs (123, 126) that potentially might have an effect on sperm activeness. A DNA microarray revealed that 559 transcripts in low-fertile bulls are dysregulated. Notably, transcripts such as PRDX6, NOS3, SOD, BAK, and BCL2L11 have been associated with fertility and are linked to the oxidation reduction process, the mediation of MMP, and apoptosis. This study provides a pathway to develop male-fertility-related markers (127).

The bull transcriptomic profile revealed that non-coding RNAs (ncRNAs) are involved in the regulation of sperm motility (128). The ncRNAs are the main regulators of spermatogenesis and male fertility but literature on lncRNAs in human oligozoospermia is scant. The sequencing data of lncRNA and mRNA from 12 human normozoospermia and oligozoospermia samples revealed the altered expression of lncRNAs (DE lncRNAs) and mRNAs (DE mRNAs) in male infertility. The Gaussian graphical model, gene ontology, and Encyclopedia of Genes and Genomes pathways were applied to identify them and investigate their possible functions. The transcriptome data showed that DE lncRNAs and DE mRNAs and their target genes were involved in the accretion of unfolded proteins in sperm ER, PERK-EIF2 pathway-induced ER stress, oxidative stress, and apoptotic sperms in individual oligozoospermia subjects. This suggests that these lncRNAs and pathways could be utilized as a therapeutic target for infertility. There is less evidence about the semen transcriptomic profile in terms of interactions with oxidative stress. More studies are required to determine whether oxidative stress is involved in male infertility problems (129).

Several RNA-seq studies have attempted to characterize the transcriptome of ejaculated spermatozoa in terms of sperm quality and fertility. Semen quality varies according to season. A total of 4,436 coding genes of moderate to high abundance have been identified in sperm RNA. The fragmentation of the transcript increased in genes associated with spermatogenesis, chromatin compaction, and fertility. The summer and winter ejaculates had different transcriptomic profiles, with 34 coding genes and 7 microRNAs showing a significantly distinct distribution. These genes were linked to oxidative stress, DNA damage, and autophagy. The annotation of the boar sperm transcriptomic profile was used to identify sperm quality markers in pigs (130). **Table 2** shows the involvement of transcriptomic factors on male infertility.

**Table 2:** Effect of transcriptomic factors on male infertility.

miRNA/transcriptomic Factors	Regulatory effect	Outcomes	References
miR-196a-2, miR-196a-5p,miR-141, miR-429, and miR-7-1-3p	Upregulation	Idiopathic male infertility	(131, 132)
miR-424	Downregulation	Idiopathic male infertility	(133)
MiR-371a-3p	Upregulation	Sperm concentration and total sperm count	(134)
piR-31068, piR-31098, piR-31925, piR-43771, and piR-43773	Differentially expressed/ downregulation	Asthenozoospermia	(135)
miR-19b and let-7a	Upregulation	Idiopathic infertility	(136)
miR-192a	Upregulation	Germ cell apoptosis	(137)

## Oxidative stress and DNA fragmentation

The overproduction of ROS may influence male infertility by interacting with different cellular components, resulting in sperm damage (138, 139). This process involves lipid peroxidation and protein oxidation through the utilization of numerous molecular mechanisms. In particular, OS induces the production of oxidized DNA adducts, such as 8-hydroxy-2’-deoxyguanosine (8OHdG) within the DNA, resulting in single- or double-strand breaks (140). Moreover, ROS stimulates caspases and nucleases that contribute to apoptotic pathways; therefore, they cause indirect damage to the sperm DNA through abortive apoptosis (141).

Presently, research measuring oxidative stress relies on estimations of intracellular ROS (using a chemiluminescence assay) (142), total antioxidant capacity (TAC) (143), malondialdehyde (144), or DNA damage (8-OHdG) (145), which have been identified as markers of OS and significant sperm damage in infertile patients (146–148). Further, sperm DNA damage impairs sperm fertility capacity and embryo development during natural conception and has been linked with assisted reproductive tools (149–151). Intriguingly, it has been noted that measurement of oxidative stress might be important for infertile subjects who can benefit from antioxidant supplementation or an alteration in lifestyle (152). These considerations show that there is a dire need to understand the correlation between seminal plasma oxidative stress and sperm DNA damage and for the development of new diagnostic methods. More recently, a novel galvanostat-based technique was used to measure OS. This technique determines the balance between oxidants and reductants in semen, which is known as the oxidation-reduction potential (ORP) (153).

Spermatozoa possess a one-base excision repair (BER) enzyme upstream during their development, which is helpful for DNA repair. This enzyme is known as 8-oxoguanine DNA glycosylase 1 (OGG1), and it assists in the release of adducts into the extracellular space through the excision of DNA base adducts (154, 155). Spermatozoa do not possess BER enzymes, such as apurinic endonuclease 1 (APE1) and x-ray repair cross-complementing protein 1 (XRCC1). For that reason, the DNA repair ability of spermatozoa is delicate, resulting in the repair of oxidized DNA base adducts, such as 8-OHdG (155). Moreover, it has been found that 8-OHdG triggers germline mutations, indirectly causing DNA damage in human spermatozoa (156).

## Male infertility preventive strategies

Male infertility is a highly concerning issue that has not received much focus in terms of better understanding its magnitude and prevalence. Several factors of male infertility are idiopathic in nature. As such, there is an emerging need to address the problem and investigate preventive strategies (157).

The following approaches should be considered for preventing male infertility problems:

1. Oxidative stress is the main cause of male infertility induction and attempts should be made to limit the production of oxidative stress. However, it should be kept in mind that some ROS production is needed to maintain male fertility.
2. The cellular mechanism involved in male infertility may provide new pathways for drug development from antioxidant compounds that are safe and secure and exert less toxic effects than commercially available classical drugs.
3. Nanoparticle-based approaches could be useful for the targeted delivery of polyphenol-derived drugs.
4. The integration of knowledge and computer science through machine learning algorithms should be adopted in male infertility diagnostic approaches, as well as in searches for targeted therapies (158).
5. An integrated AI system should assist the assessment of computerized semen analysis; AI-based applications can estimate environmental conditions and lifestyle to improve semen quality forecasts.
6. The cause of idiopathic male infertility is unknown but AI-based technology can improve the classification of fertile/infertile couples using biological and clinical signatures.
7. An attempt should be made to break down barriers linked to religious and cultural beliefs that prevent individuals from speaking openly about their infertility issues.
8. There is need of create awareness among populations so that male infertility problems can be discussed more frequently.
9. Excessive weight has been linked with reduced sperm production. Therefore, diet and daily exercise need to planned appropriately.
10. Addiction tends to influence physiological function. Addictive behavior needs to be avoided and monitored.
11. Tightly fitting clothing influences blood circulation to the genital organs and raises testicular temperature, thus disturbing semen production and decreasing fertility. Therefore, tight clothes need to be avoided.
12. Electronic gadgets that produce low levels of radiation eventually disturb sperm production. Therefore, it is better to minimize the use of these gadgets.
13. Deficiency of nutrients, particularly zinc and vitamin C, may disturb sperm production. Therefore, it is important to have a healthy and balanced diet. Supplementation can be used if the diet lacks the required nutrition.
14. Infection and inflammation may severely influence sperm production. Proper treatment following the doctor’s instructions and daily exercise boost the immune system and normalize the situation.

## Conclusions

In conclusion, we have reviewed the relationship between oxidative stress and male infertility and the involvement of proteomic studies in male infertility. We have compared the values of differential protein profiles in seminal plasma in both oxidative and physiological conditions. The proteomic profile of seminal plasma may play an important role in preventing oxidative stress, and it has been recognized as a putative marker/indicator of the prevalence of oxidative stress. With the literature in mind, the pathway analysis indicates the contribution of proteins to stress, cellular, metabolic, and regulatory pathways. The compiled studies in this Review will contribute to the exploration of the prominent causes of idiopathic male infertility. It is hoped that if male infertility is recognized at a molecular level, its diagnosis, treatment, and prevention can be improved. It was difficult to enumerate which mechanism should be targeted In normozoospermic conditions. However, this scenario is still incomplete and further research is needed to develop diagnostic assays based on methylated patterns, such as RNA and phosphorylation profiles. We further highlighted the attractiveness of sperm DNA integrity as a biomarker for unexplained infertility. In the coming years, it is expected that idiopathic fertility can be diagnosed using omics technologies.

## Author contributions

TH: conceptualization, writing—original draft preparation, MK and EM: methodology, illustration of figures and. GM and DHK, editing of manuscript, BT, funding acquisition and visualization, editing of the manuscript, YY, MIC, AF, AY, MSK editing of the manuscript. All authors contributed and approved the submitted version of manuscript.
